# Delayed Expression of PD-1 and TIGIT on HIV-Specific CD8 T Cells in Untreated HLA-B*57:01 Individuals Followed from Early Infection

**DOI:** 10.1128/JVI.02128-19

**Published:** 2020-07-01

**Authors:** Lydia Scharf, Johanna Tauriainen, Marcus Buggert, Wendy Hartogensis, David J. Nolan, Steven G. Deeks, Marco Salemi, Frederick M. Hecht, Annika C. Karlsson

**Affiliations:** a Department of Laboratory Medicine, Karolinska Institutet, Stockholm, Sweden; b Department of Medicine, Karolinska Institutet, Stockholm, Sweden; c Department of Microbiology, University of Pennsylvania, Philadelphia, Pennsylvania, USA; d Department of Medicine, University of California, San Francisco, San Francisco, California, USA; e Department of Pathology, Immunology and Laboratory Medicine, University of Florida, Gainesville, Florida, USA; f Bioinfoexperts LLC, Alachua, Florida, USA; Emory University

**Keywords:** CD8-positive T lymphocytes, HIV Gag, human HLA-B*5701 antigen, disease progression, viral load, CD4, programmed cell death protein 1, PD-1, T-cell immunoreceptor with Ig and ITIM domain, TIGIT, cellular immunity, molecular evolution, evolution, HIV-1

## Abstract

While the relationship of protective human leukocyte antigen (HLA) class I alleles and HIV progression is well defined, the interaction of HLA-mediated protection and CD8 T-cell exhaustion is less well characterized. To gain insight into the influence of HLA-B*57:01 on the deterioration of CD8 T-cell responses during HIV infection in the absence of antiretroviral treatment, we compared HLA-B*57:01-restricted HIV-specific CD8 T-cell responses to responses restricted by other HLA class I alleles longitudinally after control of peak viremia. Detailed characterization of polyfunctionality, differentiation phenotypes, transcription factor, and inhibitory receptor expression revealed progression of CD8 T-cell exhaustion over the course of the infection in both patient groups. However, early effects on the phenotype of the total CD8 T-cell population were apparent only in HLA-B*57-negative patients. The HLA-B*57:01-restricted, HIV epitope-specific CD8 T-cell responses showed beneficial functional patterns and significantly lower frequencies of inhibitory receptor expression, i.e., PD-1 and coexpression of PD-1 and TIGIT, within the first year of infection. Coexpression of PD-1 and TIGIT was correlated with clinical markers of disease progression and declining percentages of the T-bet^hi^ Eomes^dim^ CD8 T-cell population. In accordance with clinical and immunological deterioration in the HLA-B*57:01 group, the difference in PD-1 and TIGIT receptor expression did not persist to later stages of the disease.

**IMPORTANCE** Given the synergistic nature of TIGIT and PD-1, the coexpression of those inhibitory receptors should be considered when evaluating T-cell pathogenesis, developing immunomodulatory therapies or vaccines for HIV, and when using immunotherapy or vaccination for other causes in HIV-infected patients. HIV-mediated T-cell exhaustion influences the patient´s disease progression, immune system and subsequently non-AIDS complications, and efficacy of vaccinations against other pathogens. Consequently, the possibilities of interfering with exhaustion are numerous. Expanding the use of immunomodulatory therapies to include HIV treatment depends on information about possible targets and their role in the deterioration of the immune system. Furthermore, the rise of immunotherapies against cancer and elevated cancer incidence in HIV-infected patients together increase the need for detailed knowledge of T-cell exhaustion and possible interactions. A broader approach to counteract immune exhaustion to alleviate complications and improve efficacy of other vaccines also promises to increase patients’ health and quality of life.

## Abstract (precis)

Given the synergistic nature of TIGIT and PD-1, the coexpression of those inhibitory receptors should be considered when evaluating T-cell pathogenesis, developing immunomodulatory therapies or vaccines for HIV, and when using immunotherapy or vaccination for other causes in HIV-infected patients. HIV-mediated T-cell exhaustion influences the patient´s disease progression, immune system and subsequently non-AIDS complications, and efficacy of vaccinations against other pathogens. Consequently, the possibilities of interfering with exhaustion are numerous. Expanding the use of immunomodulatory therapies to include HIV treatment depends on information about possible targets and their role in the deterioration of the immune system. Furthermore, the rise of immunotherapies against cancer and elevated cancer incidence in HIV-infected patients together increase the need for detailed knowledge of T-cell exhaustion and possible interactions. A broader approach to counteract immune exhaustion to alleviate complications and improve efficacy of other vaccines also promises to increase patients’ health and quality of life.

## INTRODUCTION

There are several reasons why the correlates for broadly effective immune responses against human immunodeficiency virus type 1 (HIV) remain elusive and have yet to be harnessed by vaccination to prevent infection or cure those already infected. A great challenge in identifying correlates of a protective immune response is the genetic variation within the virus (1, 2) and the host immune system (3–6). The control of viral replication at acute infection is closely associated with the ability of the host to mount human leukocyte antigen (HLA) class I-restricted CD8 T-cell responses against viral epitopes (7, 8). In untreated HIV infection, the HLA-B*57 allele is the most consistent host factor associated with slow disease progression (9–11). In order to improve our basic understanding of CD8 T-cell responses restricted by different HLA class I alleles, studies evaluating the complex interplay between the virus genetic variability and immune responses are needed.

The mechanism behind the HLA-B*57-mediated delay in disease progression without antiretroviral treatment (ART) can differ between patients and involves both viral and host factors. Among the viral factors are the level of conservation of the HIV protein (12–15) or specific HLA-B*57-restricted epitopes targeted by the CD8 T cells (16, 17), as well as the fitness cost associated with immune escape from these responses (18). Host immunological factors include T-cell features such as CD8 T-cell cytotoxic capacity (19, 20), polyfunctionality (21), or specific effector molecules (20, 22). In a previous study, we provided insights into the roles of epitope-specific CD8 T-cell function in constraining HIV evolution in HLA-B*57:01-positive subjects with low risk compared to subjects with high risk for disease progression (9). Differences in progression in HLA-B*57:01-positive subjects were attributed to long-term maintenance of interleukin-2 (IL-2) and perforin in response to emerging viral mutants (23). These results suggest that correlates of protection involve both viral evolutionary and immunological biomarkers.

Hallmarks of persistent HIV infection include hyperactivated and exhausted T-cell responses. Typical manifestations of T-cell exhaustion are the accumulation of inhibitory receptors, loss of polyfunctionality and proliferative capacity, and transcriptional and metabolic alterations (reviewed in reference 24). Among the inhibitory receptors expressed on exhausted CD8 T cells are the following: 2B4 (CD244), CD160, killer cell lectin-like receptor G1 (KLRG-1), T-cell immunoreceptor with Ig and ITIM domains (TIGIT), and programmed cell death protein 1 (PD-1) (25, 26). It remains to be determined if HLA-B*57-mediated CD8 T-cell immune responses are associated with a differential level of exhaustion than non-HLA-B*57-mediated responses.

Through the proposed study design, we aimed to investigate whether the combined pattern of HIV evolution and HIV-specific CD8 T-cell exhaustion and functionality could explain the differing risk of HIV disease progression in HLA-B*57:01-positive and HLA-B*57:01-negative patients. The study subjects were followed during untreated HIV infection for a median of 12 estimated weeks postinfection (wpi) up to 7 years (9). Specifically, we unravel how the phenotype and expression of inhibitory molecules linked to T-cell exhaustion may affect the antiviral functional properties of the specific CD8 T-cell subsets in subjects followed from early infection. Explaining the differences between patients with or without HLA-B*57-restricted CD8 T-cell responses using this approach will have significant translational impact by providing specific correlates of protection that are essential for the development of immunotherapeutic approaches and vaccines.

## RESULTS

### Patient characteristics and differences in CD4 T-cell count and viral load.

To define the interaction of HLA-mediated protection and CD8 T-cell exhaustion after reduction of acute-phase peak viral replication, we evaluated samples post-acute-phase infection. We refer to this time (8 to 26 wpi) as early chronic infection. Twelve HIV subtype B-positive patients were monitored from early chronic infection (median, 12 wpi) up to 7 years postinfection (median, 273.5 wpi) (Table 1). Two patients received treatment for a limited time during the study, while the other 10 patients remained untreated. Six of the patients (P1 to P6) carried the HLA-B*57:01 allele (9, 23); the other six subjects (P7 to P12) did not carry any variation of the HLA-B*57 allele (Table 2).

**TABLE 1:** Detailed subject and sampling information^*a*^

Patient	Phylogenetics		T cells		HLA class I genotype
	Study period (wpi)	No. of samples	Study period (wpi)	No. of samples	
P1	13–271	4	17–271	2	A*0101, *0301; B*2705, *5701
P2	18–195	3	195	1	A*0101, *2402; B*4002, *5701
P3	13–324	5	274	1	A*2402, *3201; B*4002, *5701
P4	11–316	5	181–316	3	A*0101, *0201; B*5101, *5701
P5	13–309	5	16–309	3	A*0101, *0101; B*0801, *5701
P6	10–377	6	26–77	2	A*0301, *2402; B*3501, *5701
P7	10–321	7	10–321	7	A*02, *03; B*07, *27
P8	13–139	3	10–139	3	A*03, *11; B*07, *40/60
P9	10–155	4	10–155	4	A*02, *25; B*44, *44
P10	10–276	6	10–276	6	A*01, *30; B*08, *39
P11	8–126	3	8–126	3	A*03, *25; B*55, *40/60
P12	14–178	4	14–133	3	A*01, *02; B*07, *08

a wpi, estimated number of weeks postinfection.

**TABLE 2:** Patient characteristics

Patient	Age at start of infection (yr)	Sex	Baseline CD4 count (cells/mm^3^)	Baseline viral load (copies/ml)	CD4 slope (% change/yr)
P1^*a*^	35	Male	555	7,728	−15.7
P2	36	Male	570	4,973	−14.8
P3	40	Male	250	<50	−3.6
P4	34	Male	1,452	293	−7.0
P5^*b*^	37	Male	1,102	1,533	−6.4
P6	28	Male	833	10,861	−5.9
P7	34	Male	720	3,370	−7.3
P8	30	Male	522	124,070	−34.0
P9	50	Female	600	44,427	−17.6
P10	31	Male	620	6,070	1.9
P11	48	Male	546	6,876	−19.5
P12	42	Male	468	5,419	−10.4

a P1 received treatment for 14 months during the study period. Samples during treatment and 6 months after discontinuation were excluded.

b P5 received treatment for 2 weeks during the study period.

**FIG 1:**
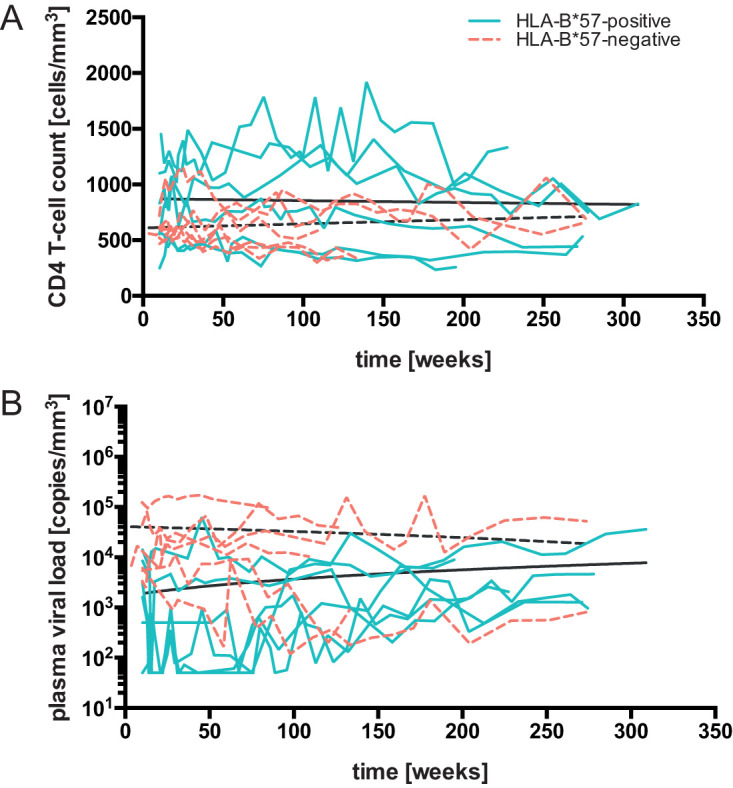
Clinical presentation and viral dynamics. (A and B) HLA-B*57:01-positive and HLA-B*57-negative patients were monitored for up to 7 years after HIV infection while they remained mainly untreated. Linear regression of CD4 T-cell counts (A) and plasma viral loads (B) of HLA-B*57:01-positive (solid turquoise lines) and HLA-B*57-negative (dashed orange lines) patients were plotted throughout the study time. Data acquired during temporary treatment intervals were excluded from the analysis.

The initial CD4 T-cell count in the HLA-B*57:01-positive group was a median 702 cells/mm^3^, and that in the HLA-B*57-negative group was a median 580 cells/mm^3^ (Table 2). The HLA-B*57:01-positive study subjects exhibited lower baseline plasma viral loads, with a median 3,253 copies/ml, than the HLA-B*57-negative group, with a median 6,473 copies/ml (Table 2). There was no significant difference in either the viral load slope or the CD4 T-cell slope between the two groups of patients, although the HLA-B*57-negative control subjects displayed a larger range of CD4 T-cell slope (−34.0 to 1.9% change/year) (Fig. 1A) than the HLA-B*57:01-positive subjects (−15.7 to −3.6% change/year) (Fig. 1B). Over the course of the study, all patients experienced disease progression (Fig. 1A and B). In addition, several HLA-B*57-negative patients started permanent treatment after 3 to 4 years and thus became ineligible for this study. Over time, CD4 T-cell counts became more similar between the two groups, but not viral load.

### Similar phylogenetic signals and sequence diversities in HIV gag p24 of HLA-B*57:01-positive and -negative subjects.

Plasma samples for phylogenetic analysis were selected from 3 to 7 time points during the study period per subject, and likelihood mapping analyses of HIV *gag* p24 sequences were performed (data not shown). Star-like signal was measured for all subjects, and no significant difference was observed between the two groups of patients (data not shown). The number of segregating and parsimony informative (Pi) sites (data not shown) did not reveal significant differences between the two groups of patients. Additionally, recombination analysis was performed for all 12 data sets (data not shown), and recombinant sequences were detected only in three sets (P1, P3, and P4). These sequences were excluded from subsequent phylogenetic analysis.

**FIG 2:**
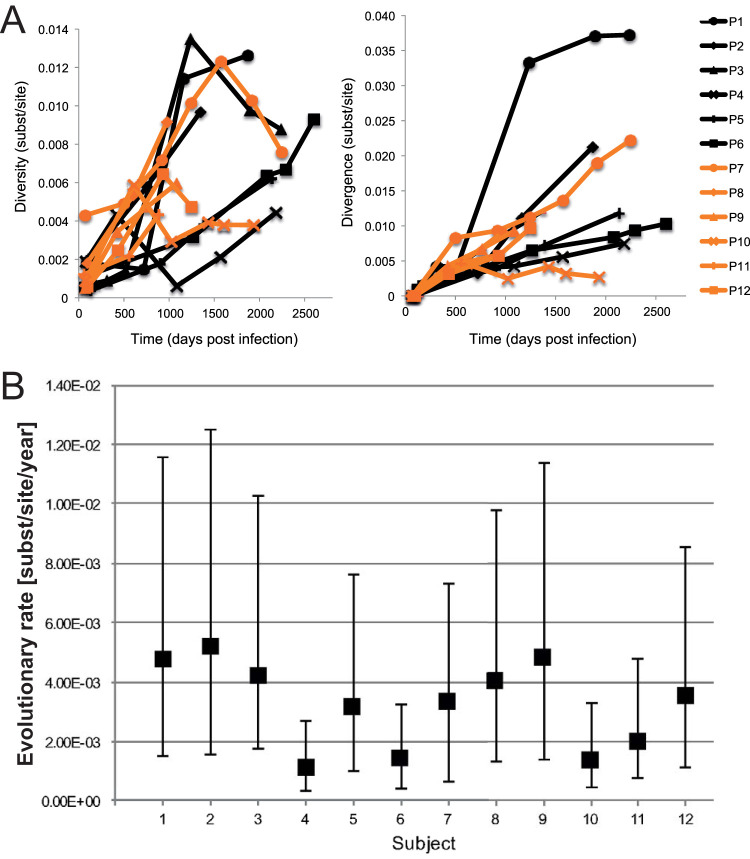
Analysis of viral evolution and functionality of HIV epitope-specific CD8 T-cell responses. (A) Longitudinal HIV p24 intrahost diversity and divergence in all 12 subjects. The six HLA-B*57:01 subjects (P1 to P6) are indicated in black, and the non-HLA-B*57 control subjects (P7 to P12) are indicated in orange. Diversity and divergence are indicated in nucleotide substitutions per site. (B) Median nucleotide substitution rate and 95% highest posterior density (HPD) intervals of HIV *gag* p24 in 12 longitudinally sampled patients. Substitution rates for six HLA-B*57:01 subjects (P1 to P6) and six non-HLA-B*57 control subjects (P7 to P12) are given in nucleotide substitutions/site/year along the *x* axis and were estimated by Bayesian inference, assuming either a strict or relaxed molecular clock depending on the best-fitting model of each subject.

In each subject-specific *gag* p24 alignment, viral diversity and divergence were measured for sequence subsets obtained at different time points (Fig. 2A and B). As expected, both the diversity and divergence increased over time (27) for all subjects, indicating that overall, significant viral evolution could be detected in both the HLA-B*57:01-positive and -negative patients (Fig. 2B) but did not differ between the groups.

### Differential frequencies of memory CD8 T-cell subsets and expression of inhibitory molecules in HLA-B*57:01-positive and HLA-B*57-negative patients.

**FIG 3:**
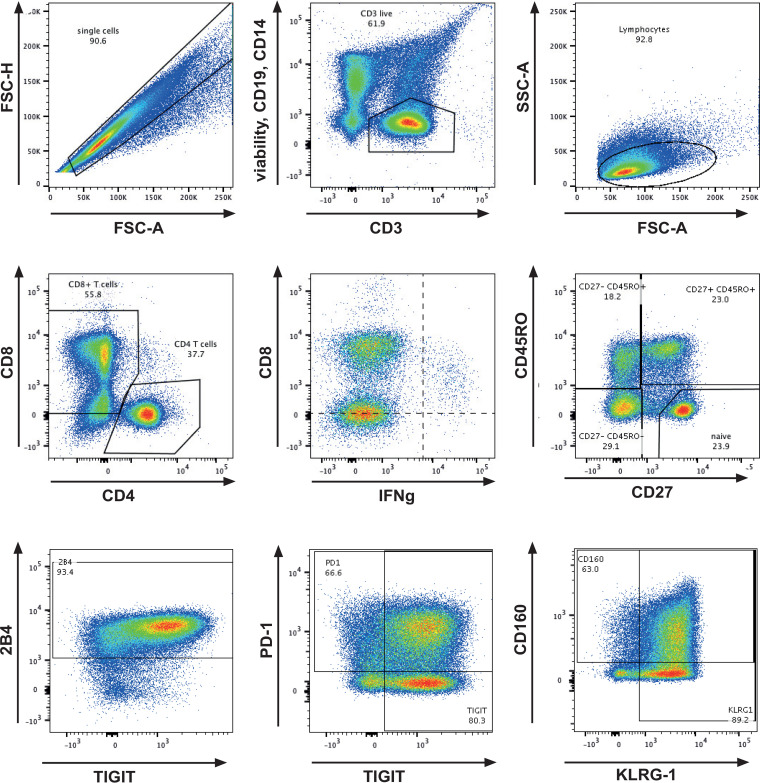
Example for gating strategy to analyze differentiation phenotypes of CD8 T cells.

We compared the differentiation profiles of total CD8 T cells between HLA-B*57-positive and -negative patients as well as HIV-negative donors. The cells were stained for CD27 and CD45RO to discriminate the following differentiation subsets (gating displayed in Fig. 3): naive (CD27^+^ CD45RO^−^), central/transitional memory (CM/TM; CD27^+^ CD45RO^+^), effector memory (EM; CD27^−^ CD45RO^+^), and effector memory reexpressing CD45RA/effector and effector-like (TEMRA/Eff; CD27^−^ CD45RO^−^) CD8 T cells.

**FIG 4:**
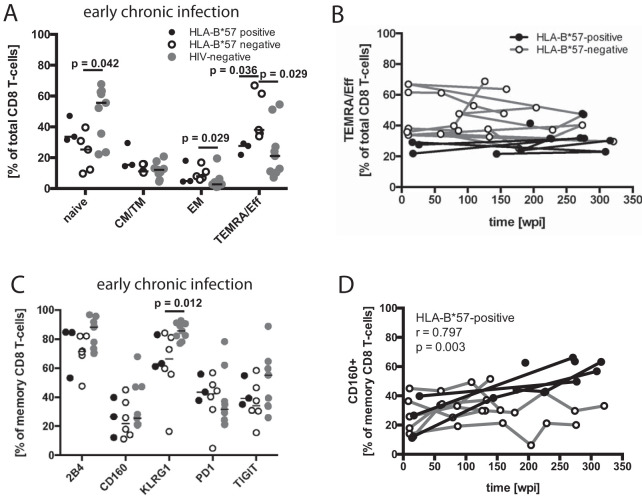
Differentiation phenotypes and inhibitory receptor expression of CD8 T cells. (A) Differentiation phenotypes of CD8 T cells from HLA-B*57:01-positive (black filled circles) and HLA-B*57-negative (empty circles) patients in early chronic infection (8 to 26 wpi), as well as HIV-negative control subjects (gray circles). *P* values are the result of Mann-Whitney tests. (B) The TEMRA subset initially differing between the patient groups in panel A was followed longitudinally throughout the study period. (C) Inhibitory receptor expression on memory CD8 T cells from HLA-B*57-negative and HLA-B*57:01-positive individuals in early chronic infection, as well as HIV-negative control subjects (gray circles). (D) CD160 expression was followed throughout the study period and analyzed for correlation with duration of HIV infection. Spearman´s rank correlation coefficient and *P* value are indicated for the study group with a significant correlation.

**FIG 5:**
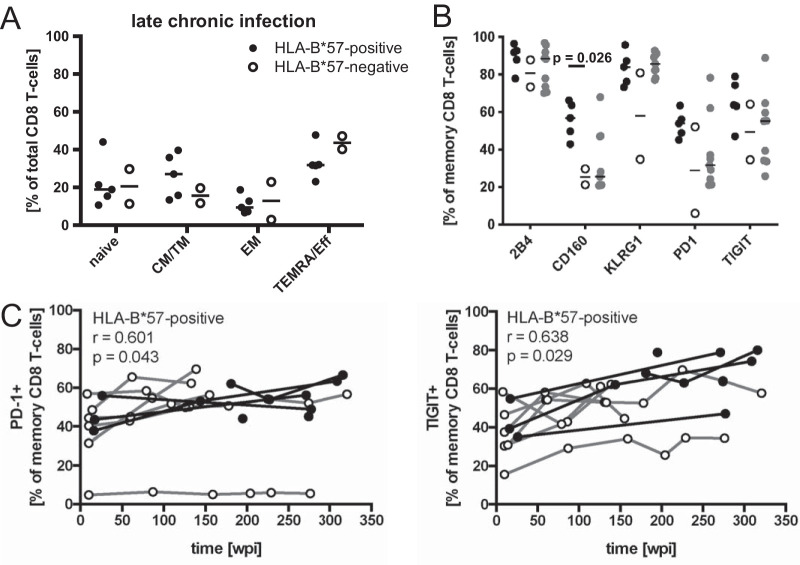
Total CD8 T-cell characterization in late chronic infection. (A) Differentiation phenotypes of CD8 T cells from HLA-B*57:01-positive (black filled circles) and HLA-B*57-negative (empty circles) patients in late chronic infection (155 to 309 wpi). (B) Frequency of inhibitory receptor expression on memory CD8 T cells in late chronic infection. (C) Frequencies of PD-1 and TIGIT expression by memory CD8 T cells were followed over the study period. Spearman´s rank correlation coefficient and *P* value are indicated for the patient group with significant correlation.

A cross-sectional comparison in early chronic infection (8 to 26 wpi) revealed that the frequency of TEMRA/Eff CD8 T cells was higher among HLA-B*57-negative patients (*P* = 0.036) than HLA-B*57-positive patients (Fig. 4A). This difference was maintained when the available longitudinal data points up to week 150 were plotted (Fig. 4B) but not in a cross-sectional comparison during late chronic infection (155 to 309 wpi) (Fig. 5A).

In comparison to HIV-negative control subjects, HLA-B*57-negative patients showed higher frequencies of EM (*P* = 0.029) and TEMRA/Eff CD8 T cells (*P* = 0.029) as well as lower frequencies of naive CD8 T cells (Fig. 4A). We did not observe significant differences in the frequencies of CD8 T cells of any differentiation state between HLA-B*57-positive HIV-infected patients and HIV-negative control subjects (Fig. 4A).

The frequencies of inhibitory receptor expression on total memory CD8 T cells were then evaluated (Fig. 4C). In early chronic infection, all receptors were expressed at highly similar levels between the groups. In late chronic infection (155 to 309 wpi), HLA-B*57:01-positive patients had a higher frequency of CD160-expressing cells than HLA-B*57-negative patients (*P* = 0.026) (Fig. 5B). Longitudinal data of CD160 expression among memory CD8 T cells in HLA-B*57-positive patients confirmed an increasing frequency (*r* = 0.797, *P* = 0.003) over the duration of the infection, which was not observed in HLA-B*57-negative patients (Fig. 4D). Similarly, the frequency of PD-1 and TIGIT expression among CD8 memory T-cells increased in HLA-B*57-positive patients over time but not in the HLA-B*57-negative group (Fig. 5C), despite the lack of significant differences in cross-sectional comparisons during either early (Fig. 4C) or late (Fig. 5B) chronic infection.

In comparison to HIV-negative control subjects, HLA-B*57-negative patients during early chronic infection had lower frequencies of KLRG-1-expressing memory CD8 T cells (*P* = 0.012) (Fig. 4C) and HLA-B*57-positive patients during late chronic infection had higher proportions of CD160-expressing cells (*P* = 0.042) (Fig. 5B).

In summary, HLA-B*57-negative patients displayed altered patterns of CD8 T-cell differentiation in comparison to HIV-negative subjects within the first year of infection, patterns which were not apparent in HLA-B*57-positive patients. The lower frequency of the TEMRA/Eff subset distinguished HLA-B*57-positive from HLA-B*57-negative patients. Furthermore, the frequency of memory CD8 T cells expressing CD160, PD-1, and TIGIT increased longitudinally in HLA-B*57-positive but not in HLA-B*57-negative patients.

### Impairment of functional features is aggravated in chronic infection (3 to 6 years after infection).

CD8 T-cell exhaustion is a phenomenon that affects HIV-specific T-cell responses more severely than the total or cytomegalovirus (CMV)-specific CD8 T-cell population of HIV-infected patients (26). Here, we aimed to compare autologous HIV epitope-specific CD8 T-cell responses restricted by HLA-B*57:01 to responses restricted by other alleles in HLA-B*57-positive and HLA-B*57-negative study subjects. To this end, we stimulated peripheral blood mononuclear cells (PBMCs) with optimal CD8 T-cell epitopes corresponding to the patient’s autologous HIV sequences and HLA alleles and identified responding CD8 memory T cells by their expression of CD107a, gamma interferon (IFN-γ), tumor necrosis factor (TNF), and/or CD107a. We then assessed the frequency of responding cells among CD8 memory T cells, as well as the proportion of functional markers and their combinations.

The HLA-restricted immune responses were characterized in a cross-sectional manner during early (8 to 26 wpi) and late (195 to 309 wpi) chronic HIV infection (Table 3), as well as longitudinally (Table 4). For cross-sectional comparisons, we selected the one or two strongest HIV epitope-specific CD8 T-cell responses per patient and time point for analysis. Responses detected against more than one variant of a specific epitope (i.e., corresponding to amino acid mutations), which could be reflective of cross-reactive T cells, were not included. For longitudinal analysis, the epitope-specific responses were selected in the same manner over all available time points but also included an additional response for patient 7 (P7) at the earliest time point (10 wpi) for improved longitudinal follow-up of a response being maintained at later time points. To avoid strong bias toward patients represented by a higher number of data points (epitope-specific responses), we used mixed-effects models for longitudinal analysis.

**TABLE 3:** Samples, clinical characteristics, and epitope sequences for cross-sectional comparisons of HLA-restricted CD8 T-cell responses in early and late chronic HIV infection

Chronic infection type and patient	HLA-B*57	No. of wpi^*a*^	Viral load (baseline DNA copies/ml)	CD4 count (cells/mm^3^)	Epitope
Early					
P1	pos	17	56	1,078	TSTLQEQIGW
P5	pos	16	724	1,216	TSTLQEQIGW
P6	pos	26	9,266	800	KAFSPEVIPMF
P6	pos	26	9,266	800	ISPRTLNAW
P7	neg	10	3,370	720	GPSHKARVL
P7	neg	10	3,370	720	KRWIVMGLNK
P8	neg	10	124,070	522	RLRPGGKKR
P9	neg	10	44,427	600	ETINEEAAEW
P10	neg	10	6,074	620	TPQDLNTML
P11	neg	8	16,910	546	ETINEEAAEW
P11	neg	8	16,910	546	RLRPGGKKK
P12	neg	14		507	EVYKKWII
Late					
P1	pos	271	1,276	442	KAFSPEIIPMF
P1	pos	271	1,276	442	LSPRTLNAW
P2	pos	195	8,976	257	ISPRTLNAW
P2	pos	195	8,976	257	KAFSPEVIPMF
P3	pos	274	972	533	KAFSPEVIPMF
P3	pos	274	972	533	HTQGYFPDWQ
P4	pos	227	2,069	1,333	KAFSPEVIPMF
P4	pos	227	2,069	1,333	QASQEVKNW
P5	pos	309	35,941	826	QASQEVKNW
P5	pos	309	35,941	826	KAFSPEVIPMF
P6	pos	277	4,631	748	KAFSPEVIPMF
P6	pos	277	4,631	748	LSPRTLNAW
P7	neg	274	52,450	648	KRWIVMGLNK
P9	neg	155	33,418	297	ETINEEAAEW
P10	neg	229	550	704	TPQDLNTML

a wpi, estimated number of weeks postinfection.

**TABLE 4:** Epitope sequences and samples for longitudinal analysis

Patient	HLA-B*57	Epitope	Time point(s) (wpi^*c*^)
P1	pos	TSTLQEQIGW	17
		LSPRTLNAW	271
		KAFSPEIIPMF	271
P2	pos	ISPRTLNAW	195
		KAFSPEVIPMF	195
P3	pos	HTQGYFPDWQ	274
		KAFSPEVIPMF	274
P4	pos	KAFSPEVIPMF	181, 227, 316
		QASQEVKNW	181, 227, 316
P5	pos	TSTLQEQIGW	16
		KAFSPEVIPMF	144, 309
		ISPRTLNAW	144
		QASQEVKNW	309
P6	pos	ISPRTLNAW	26
		KAFSPEVIPMF	26, 277
		LSPRTLNAW	277^*a*^
P7	neg	KRWIILGLNK	10, 59, 131, 178, 225, 274, 321
		GPGHKARVL	10, 59, 131, 178
		GPSHKARVL	10
P8	neg	RLRPGGKKR	10, 86, 139
P9	neg	ETINEEAAEW	10^*a*^, 60, 109, 155
P10	neg	TPQDLNTML	10^*a*^, 87, 159^*b*^, 204^*a*^, 229, 276
P11	neg	RLRPGGKKK	8, 79, 126
		ETINEEAAEW	8
P12	neg	EVYKKWII	14, 62, 133

a Only data from functional panel available.

b Only data from exhaustion panel available.

b wpi, estimated number of weeks postinfection.

**FIG 6:**
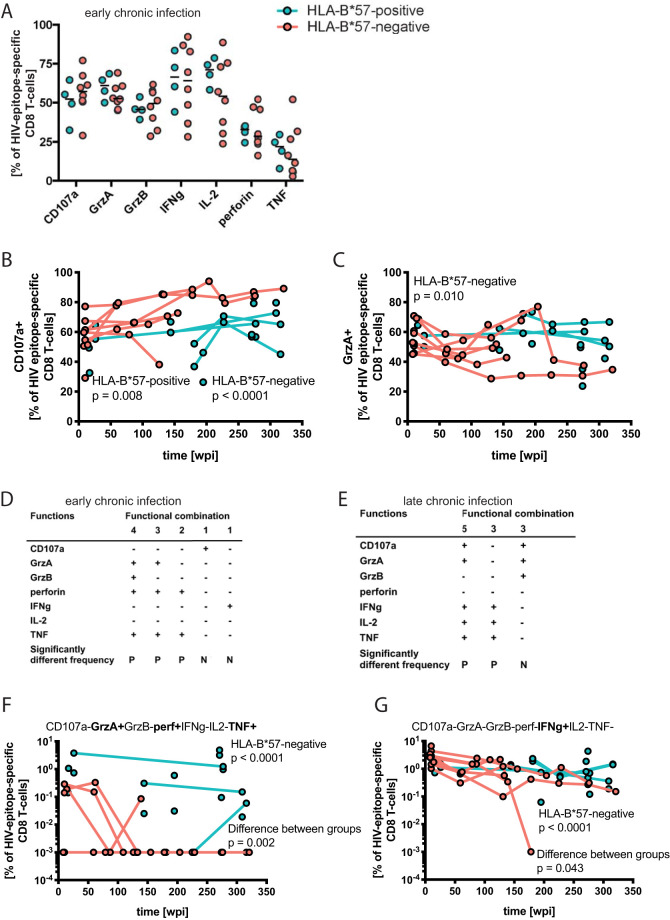
Functional features and transcription factor profile of HIV epitope-specific CD8 T-cell responses. (A) Effector molecule expression of HIV epitope-specific HLA-restricted CD8 T-cell responses. Per sample, the properties of the strongest autologous HIV Gag-specific CD8 T-cell responses as detected by the production of gamma interferon (IFN-γ), IL-2, tumor necrosis factor (TNF), and/or CD107a were identified and used in the comparison. Epitopes are defined in Table 3. Data from early chronic infection are depicted with HLA-B*57:01-restricted responses as turquoise filled circles, and responses restricted by other HLA class I alleles are depicted as orange filled circles. The plot shows individual values and the median. (B and C) The frequencies of effector molecule expression among responding cells were followed longitudinally. Depicted are functions showing significant correlation with the duration of HIV infection (significance was determined using a mixed-effects model; *P* value and study group are indicated in the panels). (D and E) Polyfunctionalities were compared between HLA-B*57:01-restricted responses and responses restricted by other HLA alleles. The simultaneous expression of CD107a, granzyme A (GrzA), GrzB, perforin, IFN-γ, IL-2, and TNF on a single-cell level was used for the Student´s *t* test provided in the SPICE software. Expression patterns that differed between the groups during early or late chronic infection are depicted. P, Significantly more frequent population in the HLA-B*57:01-restricted responses; N, significantly more frequent population in the responses restricted by other alleles. (F and G) Functional combinations from panels D and E with significant longitudinal changes are shown. The 10^−3^ values are arbitrary in order to visualize the absence of the indicated functional combination among the respective HIV-specific responses on a logarithmic axis. Analysis was done with original values and the Mann-Whitney test (rank test), rendering the absolute value inconsequential. The significance of correlation with duration of infection and differences between groups were determined using a mixed-effects model; *P* value and study group are indicated in the panels.

We found no significant differences in quantity of HIV-specific cells as a percentage of CD8 memory T cells responding to peptide stimulation (data not shown). When the data on individual functional markers were compared in a cross-sectional manner during early or late chronic infection, a high variability between the individual responses dominated over any differences between the patient groups (Fig. 6A). Differences, as seen in the higher frequency of IL-2-, GrzA-, or TNF-producing HLA-B*57-restricted cells and CD107a-producing cells restricted by other alleles, did not reach significance due to the small sample size. However, longitudinal analyses revealed sustained higher frequency of CD107a-producing CD8 T cells among HLA-B*57-negative subjects as well as an increase in the proportion of cells expressing CD107a among both HLA-B*57-restricted (estimated unit change per additional wpi, 0.064; *P* = 0.008) and responses restricted by other alleles (estimated unit change per additional wpi, 0.100; *P* < 0.0001) (Fig. 6B; Table 5). Among non-HLA-B*57-restricted responses, the frequency of GrzA-expressing cells decreased (estimated unit change per additional wpi, −0.058; *P* = 0.010) (Fig. 6C; Table 5), while the respective counterparts showed no significant change with duration of infection.

**TABLE 5:** Mixed-effects models reported as unit change or fold change^*a*^

Figure	*y*-axis term	*x* axis	Change	Group	Estimate of change (95% CI)	*P* value (within-group change)^*b*^	*P* value (between groups)
6B	CD107a^+^	Time (wpi)	Unit change per additional wpi	B57 negative	0.100 (0.055 to 0.145)	**<0.0001**	0.27
				B57 positive	0 .0635576 (0.016 to 0.111)	**0.008**	
6C	GrzA^+^	Time (wpi)	Unit change per additional wpi	B57 negative	−0.058 (−0.102 to −0.014)	**0.010**	0.52
				B57 positive	−0.037 (−0.084 to +0.010)	0.12	
6F^*c*^	CD107a^−^ GrzA^+^ GrzB^−^ IFN-γ^−^ IL-2^−^ Perforin^+^ TNF^+^	Time (wpi)	Percent change per additional wpi	B57 negative	−1.6% (−2.2% to −1.1%)	**<0.0001**	**0.002**
				B57 positive	−0.3% (−0.9% to +0.3%)	0.27	
6G	CD107a^−^ GrzA^−^ GrzB^−^ IFN-γ^+^ IL-2^−^ Perforin^−^ TNF^−^	Time (wpi)	Percent change per additional wpi	B57 negative	−0.9% (−1.2% to −0.5%)	**<0.0001**	**0.043**
				B57 positive	−0.3% (−0.7% to +0.1%)	0.13	
7E	PD1^+^ TIGIT^+^	Time (wpi)	Unit change per additional wpi	B57 negative	0.040 (−0.015 to +0.095)	0.15	0.091
				B57 positive	0.108 (0.052 to 0.165)	**<0.001**	
7F	PD1^+^ TIGIT^+^	Viral load [log_10_(copies/ml)]	Unit change for each 10-fold difference in viral load	B57 negative	5.805 (−2.105 to +13.715)	0.15	**0.031**
				B57 positive	19.504 (9.946 to 29.061)	**<0.001**	
7G	PD1^+^ TIGIT^+^	CD4 count [log_2_(cells/μl)]	Unit change per 2-fold increase in CD4 count	B57 negative	−6.634 (−16.999 to +3.731)	0.21	0.17
				B57 positive	−17.109 (−28.032 to −6.186)	**0.002**	
7H	PD1^+^ TIGIT^+^	CD4%	Unit change per CD4 percentage point increase	B57 negative	−0.739 (−1.463 to −0.015)	**0.046**	0.25
				B57 positive	−1.338 (−2.058 to −0.619)	**<0.001**	
7I	PD1^+^ TIGIT^+^	CD4/CD8 ratio	Unit change per unit increase in CD4/CD8 ratio	B57 negative	−22.956 (−43.032 to −2.881)	**0.025**	0.41
				B57 positive	−34.071 (−50.909 to −17.233)	**<0.001**	
8B	2B4^+^	Time (wpi)	Unit change per additional wpi	B57 negative	0.118 (0.077 to 0.159)	**<0.0001**	0.83
				B57 positive	0.124 (0.084 to 0.165)	**<0.0001**	
8B	CD160^+^	Time (wpi)	Unit change per additional wpi	B57 negative	0.018 (−0.015 to +0.052)	0.28	**0.002**
				B57 positive	0.093 (0.060 to 0.125)	**<0.0001**	
8B	KLRG1^+^	Time (wpi)	Unit change per additional wpi	B57 negative	0.102 (0.060 to 0.144)	**<0.0001**	0.33
				B57 positive	0.132 (0.090 to 0.173)	**<0.0001**	
8B	PD1^+^	Time (wpi)	Unit change per additional wpi	B57 negative	0.040 (−0.013 to +0.093)	0.14	0.099
				B57 positive	0.102 (0.050 to 0.154)	**0.0001**	
8B	TIGIT^+^	Time (wpi)	Unit change per additional wpi	B57 negative	0.041 (−0.005 to +0.087)	0.079	**0.035**
				B57 positive	0.110 (0.065 to 0.155)	**<0.0001**	
9B	T-bet^hi^ Eomes^dim^	Time (wpi)	Percent change per additional wpi	B57 negative	−0.1% (−0.3% to +0.1%)	0.18	0.097
				B57 positive	−0.4% (−0.6% to −0.2%)	**<0.001**	
9C	T-bet^dim^ Eomes^hi^	Time (wpi)	Percent change per additional wpi	B57 negative	+0.1% (−0.1% to +0.2%)	0.43	0.16
				B57 positive	+0.2% (+0.1% to +0.4%)	**0.007**	
9D	T-bet^−^ Eomes^−^	Time (wpi)	Percent change per additional wpi	B57 negative	0.0% (−0.2% to +0.3%)	0.84	**0.002**
				B57 positive	−0.5% (−0.8% to −0.3%)	**<0.001**	

a The null hypothesis corresponds to 0 (unit change) or 0% (percent change). B57, HLA-B*57; CI, confidence interval. Boldface indicates statistical significance (*P* < 0.05).

b H_0_, the null hypothesis; no change.

c A zero-inflated negative binomial (ZINB) regression was used to account for the high number of absent cell populations (0 values).

Polyfunctionality, defined as the simultaneous expression of multiple effector molecules, was evaluated during early and late chronic infection. The cross-sectional comparison of HLA-B*57:01-restricted to non-HLA-B*57-restricted HIV-specific CD8 T-cell responses revealed no differences in the permutation test (data not shown). We did, however, find differences in specific populations among responding cells. In early chronic infection, five cell populations with distinct functional assets were found at significantly different frequencies between the patient groups (Fig. 6D and E). Three cell populations, expressing two to four effector molecules simultaneously (GrzA/GrzB/perforin/TNF, GrzA/perforin/TNF, and perforin/TNF) were more frequently detected in responses to HLA-B*57:01-restricted epitopes, while those more frequent in non-HLA-B*57-restricted responses were singly positive for either CD107a or IFN-γ (Fig. 6D). Longitudinal analysis of those cell populations revealed decreasing frequencies of GrzA/perforin/TNF triple-positive cells (percent change per additional wpi, −1.6%; *P* < 0.0001) as well as IFN-γ single-positive cells (percent change per additional wpi, −0.9%; *P* < 0.0001) among non-HLA-B*57-restricted responses (Fig. 6F and G; Table 5) but not among HLA-B*57-restricted responses. This difference in association with duration of HIV infection is significantly different between the groups (*P* = 0.002 for GrzA/perforin/TNF triple-positive cells; *P* = 0.043 for the IFN-γ single-positive cell population) (Table 5).

In late chronic infection, HLA-B*57:01-restricted responses harbored a higher proportion of cell populations positive for three or five markers (CD107a/GrzA/IFN-γ/IL-2/TNF and IFN-γ/IL-2/TNF), while responses restricted by other alleles were higher in a cell population positive for CD107a, GrzA, and GrzB (Fig. 6F).

In summary, our results indicate differences in the polyfunctionality pattern of responding HIV epitope-specific CD8 T cells restricted by HLA-B*57:01 or other HLA alleles. The HLA-B*57-restricted responses displayed higher frequencies of granzyme A, and of perforin together with TNF, but constantly fewer CD107a-positive cells than the non-HLA-B*57-restricted responses.

### Differential TIGIT and PD-1 expression of HIV-specific CD8 T-cell responses is apparent within a few months after infection.

To gain insight into the exhaustion of HIV epitope-specific CD8 T-cell responses restricted by either HLA-B*57:01 or other HLA class I alleles, we identified responding CD8 memory T cells by their expression of CD107a and/or IFN-γ and assessed their expression of the inhibitory receptors 2B4, CD160, KLRG-1, PD-1, and TIGIT.

**FIG 7:**
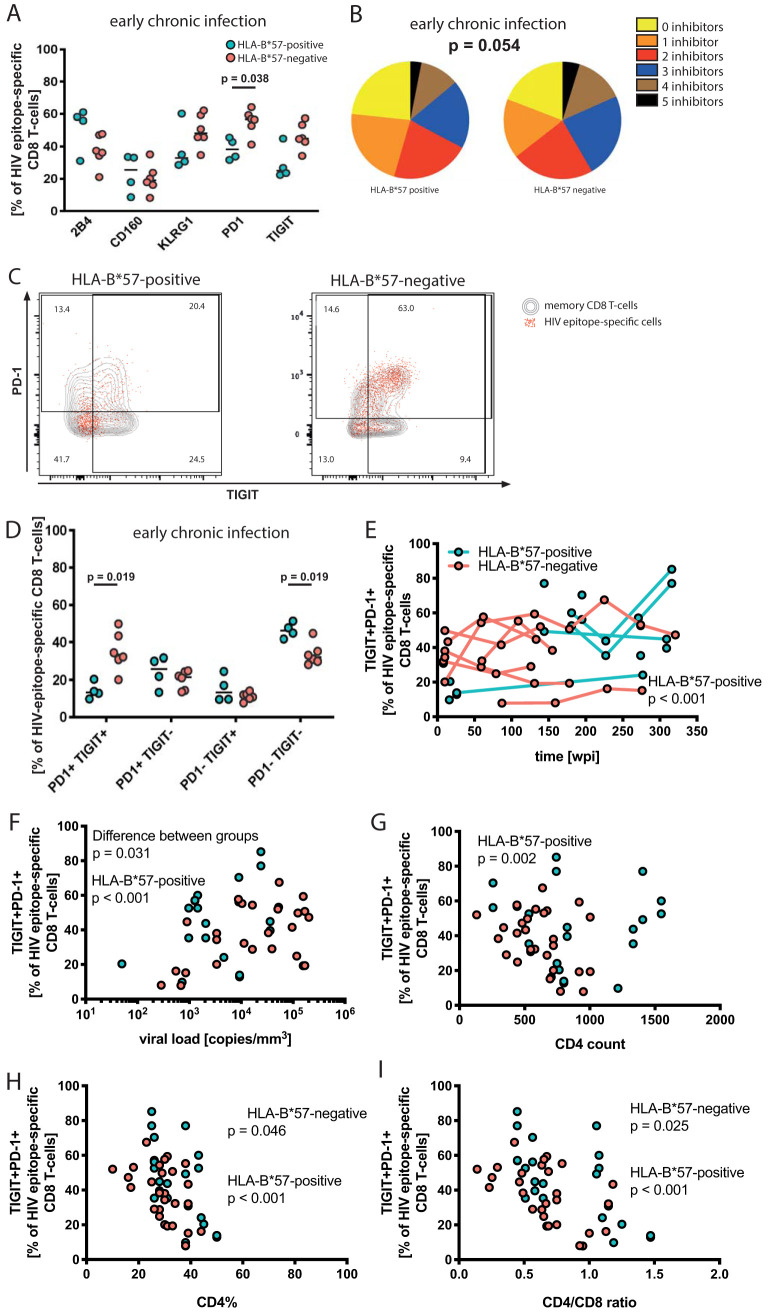
Inhibitory receptor expression of epitope-specific CD8 T cells. (A) Expression of the inhibitory receptors 2B4, CD160, KLRG-1, PD-1, and TIGIT on HIV epitope-specific CD8 T cells. HLA-B*57:01-restricted responses are depicted as turquoise filled circles; responses restricted by other HLA class I alleles are depicted as orange filled circles. The plot shows individual values and the median; *P* values are the result of the Mann-Whitney test. (B) Combinations of inhibitory receptors on the single-cell level were compared using a permutation test. Frequencies of individual expression patterns were grouped by the number of simultaneously expressed inhibitory receptors on a single-cell level and visualized with no inhibitor (yellow) to up to 5 inhibitors (black). (C) Examples of PD-1 and TIGIT expression of HIV epitope-specific responses. Contour plots in the background depict memory CD8 T cells; overlaid dot plots are HIV epitope-specific CD8 T-cell responses. TIGIT expression is depicted along the *x* axis, and PD-1 expression is depicted along the *y* axis. (D) PD-1/TIGIT coexpression of HIV epitope-specific CD8 T cells in early chronic infection. (E to I) Frequency of PD-1/TIGIT coexpression plotted against duration of viral infection (E), viral load (F), CD4 count (G), CD4% (H), and CD4/CD8 ratio (I). The significance of correlations and the difference between groups were determined using a mixed-effects model; *P* value and study group are indicated in the panels.

**FIG 8:**
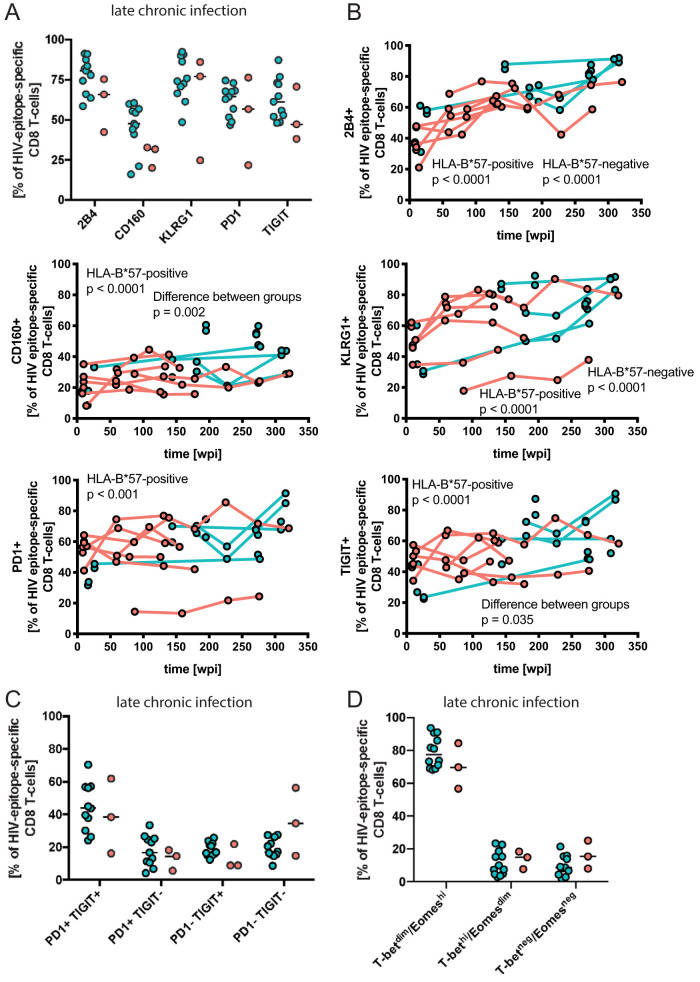
Inhibitory receptor expression and transcriptional profile of HIV epitope-specific CD8 T cells in late chronic infection and longitudinally. (A) Frequency of inhibitory receptor expression in late chronic infection. (B) Frequencies of inhibitory receptor-expressing cells were followed longitudinally. Spearman´s rank correlation coefficient, *P* value, and patient group are indicated for significant correlations. (C) Frequency of combined TIGIT and PD-1 expression in late chronic infection. (D) Transcriptional profiles of HIV epitope-specific CD8 T cells in late chronic infection.

In early chronic infection, we found lower percentages of cells expressing PD-1 (*P* = 0.038) and a trend toward lower frequencies of TIGIT expression (*P* = 0.067) among HLA-B*57:01-restricted responses than among the responses restricted by other HLA alleles (Fig. 7A). This difference was not apparent in late infection (Fig. 8A). In agreement with the blunted difference over time, the frequency of 2B4 (*P* < 0.0001), CD160 (*P* < 0.0001), KLRG-1 (*P* < 0.0001), PD-1 (*P* < 0.001), and TIGIT (*P* < 0.0001) expression among HLA-B*57-restricted CD8 T-cell responses increased over time, while only 2B4 (*P* < 0.0001) and KLRG-1 (*P* < 0.0001) expression significantly correlated with time in responses restricted by other alleles (Fig. 8B; Table 5). The frequency of responding HLA-B*57-restricted CD8 T cells expressing CD160 (*P* = 0.002) and TIGIT (*P* = 0.035) increased more rapidly over time than the level of expression on responding T cells restricted by other alleles (Table 5).

The permutation test comparing HLA-B*57:01-restricted HIV-specific CD8 T-cell responses to responses restricted by other alleles revealed a trend implying differences in early (*P* = 0.054) but not late (*P* = 0.750) chronic infection (Fig. 7B). In early chronic infection, all cell populations differing between the groups coexpressed TIGIT and PD-1 together with one to three additional inhibitory receptors or did not express TIGIT or PD-1. Consequently, we analyzed the frequencies of TIGIT and/or PD-1 expression on HIV-specific responses in detail (Fig. 7C). Cross-sectional comparisons of data from early chronic infection confirmed a significantly lower frequency of cells coexpressing TIGIT and PD-1 (*P* = 0.019) and a higher frequency of TIGIT and PD-1 double-negative cells (*P* = 0.019) among HLA-B*57:01-restricted responses (Fig. 7D). The frequencies of the single-positive cell populations (TIGIT^+^ PD-1^−^ or TIGIT^−^ PD-1^+^) were similar between the groups (Fig. 7D). In agreement with the permutation test, the expression of TIGIT and PD-1 double-positive cells did not differ significantly between the patient groups at late chronic infection (Fig. 8C). Longitudinally, only HLA-B*57-restricted responses expressing both TIGIT and PD-1 significantly correlated with time of infection (unit change per additional wpi, 0.108; *P* < 0.001), but the difference between the groups did not reach significance (*P* = 0.091) (Fig. 7E; Table 5).

Given the close relationship of CD8 T-cell exhaustion and antigen levels, we investigated the correlation between the frequency of TIGIT and PD-1 double-positive cells and clinical parameters. While we found that TIGIT and PD-1 double-positive cells correlated with viral load for HIV-specific HLA-B*57-restricted responses (estimated unit change for each 10-fold difference in viral load, 19.504; *P* < 0.001), responses restricted by other alleles showed no significant correlation (Fig. 7F; Table 5). The correlation with viral load was significantly different between the groups (*P* = 0.031). Only HLA-B*57-restricted responses showed significant correlation between the frequency of TIGIT and PD-1 double-positive cells and CD4 count (estimated unit change per 2-fold increase in CD4 count, −17.109; *P* = 0.002) (Fig. 7G; Table 5). Both groups showed inverse correlations with the percentage of CD4 T-cells among lymphocytes (CD4%; *P* < 0.001 for B*57-restricted responses, *P* = 0.046 for other responses) and CD4/CD8 ratio (*P* < 0.0001 for B*57-restricted responses, *P* = 0.025 for other responses) (Fig. 7H and I; Table 5).

In summary, the lower frequency of cells coexpressing TIGIT and PD-1 among HLA-restricted HIV-specific responses within the first year of HIV infection distinguished HLA-B*57-positive from HLA-B*57-negative patients.

### Similar transcription factor profiles in both patient groups.

The transcription factors eomesodermin (Eomes) and T-bet are fundamental for CD8 T cells: T-bet correlates with effector functions, and Eomes is associated with the differentiation and long-term survival of memory cells (28–30). Their expression levels seem to some degree mutually exclusive, as CD8 T cells high in one of the transcription factors show only intermediate expression of the other, leading to the distinction of T-bet^hi^ Eomes^dim^ and T-bet^dim^ Eomes^hi^ CD8 T-cell populations.

**FIG 9:**
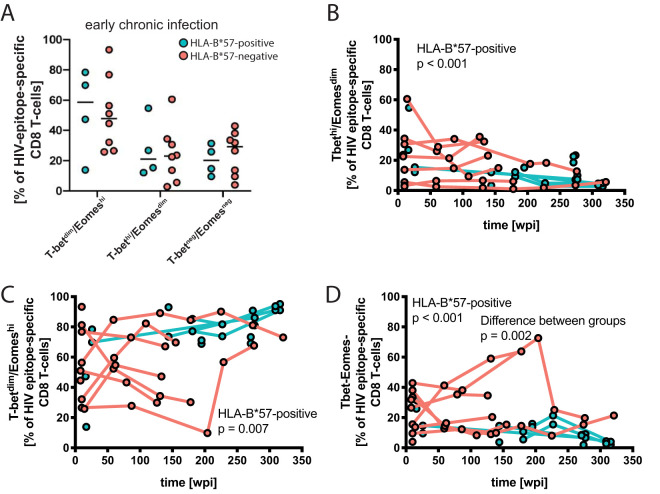
Transcriptional profile of HIV epitope-specific CD8 T cells. (A) Frequency of T-bet^dim^ Eomes^hi^, T-bet^hi^ Eomes^dim^, and T-bet^−^ Eomes^−^ cell populations among HIV epitope-specific CD8 T cells during early chronic infection. (B) Proportions of cells with the analyzed transcription factor profiles were followed longitudinally. Significant correlations with duration of infection were determined using a mixed-effects model; *P* value and study group are indicated in the panels.

Cross-sectional comparisons of the transcription factor profiles did not reveal any significant differences between the patient groups in early or late chronic infection (Fig. 9A). However, consistent with previous findings (25), we found declining percentages of the T-bet^hi^ Eomes^dim^ CD8 T-cell population (percent change per additional wpi, −0.4%; *P* < 0.001) and a corresponding increase in T-bet^dim^ Eomes^hi^ cells over time (percent change per additional wpi, 0.2%; *P* = 0.007) in our HLA-B*57-positive study subjects (Fig. 9B and C; Table 5). Similar changes in the HLA-B*57-negative patients were not statistically significant, consistent with the greater individual differences and genetic heterogeneity in this group. A third cell population negative for both T-bet and Eomes showed a decrease over the study duration in HLA-B*57-positive patients (percent change per additional wpi, −0.5%; *P* < 0.001) (Fig. 9D; Table 5) and no change over time among HLA-B*57-negative patients (Fig. 9D; Table 5). This discrepancy between the groups reached statistical significance (*P* = 0.002).

## DISCUSSION

To determine the expression dynamics of markers associated with antigen-specific CD8 T-cell exhaustion linked to HLA restriction in untreated HIV infection, we analyzed HIV-specific responses restricted by HLA-B*57:01 and other HLA class I alleles longitudinally from early chronic infection. Since disease protection is linked to CD8 T cells targeting the more conserved HIV Gag protein (12, 13, 31) and distinct epitopes within the Nef protein (23), as opposed to other HIV proteins, we used sequence data to identify and characterize CD8 T-cell responses against autologous HIV Gag and Nef epitopes. The patients, positive or negative for HLA-B*57:01, were monitored regarding clinical and immunological measures while remaining off ART.

The close link of T-cell activation and exhaustion, e.g., upregulation of inhibitory receptors and changes in functionality, urged us to use samples retrieved after the conclusion of the acute stage of infection and peak viremia. At this stage, the vast majority of affected T cells have expectedly proceeded from their prime effector stages into various degrees of exhaustion due to constant antigen exposure. Furthermore, to observe effects related to HLA restriction rather than features of viral control phenotypes, both patient groups comprised individuals with progressing disease and baseline CD4 T-cell counts above as well as below a threshold of 750 cells/mm^3^. As a group, the HLA-B*57:01-positive patients showed initially higher CD4 T-cell counts, which were similar after 3 years of infection. Although the viral load increased at a similar rate in the two groups, it remained lower in the HLA-B*57:01-positive subjects throughout the study period. When the study subjects progressed, they initiated ART and were excluded from this study; the threshold for initiating ART during this study was typically 350 cells/mm^3^. Because we studied participants who had specimens available for a significant duration of time without the initiation of ART, the HLA-B*57-negative participants are likely not representative of all persons with HIV who are HLA-B*57 negative but may be enriched for other factors that lead to slow disease progression. Still, longitudinal data of untreated HIV infection provide unique insight into disease mechanisms of the natural course of disease.

In agreement with the increasing viral loads, analysis of the viral diversity revealed comparable evolutionary rates with great variability between individual subjects regardless of the presence or absence of HLA-B*57:01. Importantly, it is known that a specific mutation linked to HLA-B*57:01-restricted HIV Gag-specific CD8 T-cell responses can hamper the HIV replicative capacity (16, 19). Such differences in early infection can later be abolished by compensatory mutations (32). Maintained viral control has also been linked by us and others to the ability to recognize mutant epitopes with maintained functional capacity (16, 19, 23, 33). Our results are also in line with earlier studies showing similar rates of viral evolution in epitope sequences from slow progressors and elite controllers in comparison to progressing HLA-B*57:01-positive subjects (16, 33). Still, when conducting more in-depth analysis of viral evolution and risk of progression, we have previously shown in the HLA-B*57:01-positive subjects that low-risk progressors, defined by a CD4 count above 750 cells/mm^3^ at baseline, display a lower HIV synonymous rate linked to the replicative capacity of the virus (34). As viral evolution was not the main scope of this study, further analysis was not conducted.

When comparing immunological features, we observed a higher overall proportion of CD45RO^−^ CD27^−^ TEMRA/Eff CD8 T cells in the HLA-B*57-negative patients during early chronic infection. Using tetramers, Ogg et al. has previously linked an increase of this phenotype among epitope-specific CD8 T-cells to disease progression in untreated subjects followed from primary infection (31), although this was not confirmed by other studies (35). In our study, the differentiation phenotypes were comparable between the patient groups throughout the study period. However, in comparison to HIV-negative control subjects, HLA-B*57-negative patients displayed higher frequencies of EM and TEMRA/Eff and lower frequencies of naive CD8 T cells already in early chronic infection. This suggests an effect on the bulk T-cell population, either advancing the T-cell pool toward later differentiation stages or constraining maintenance or generation of naive T cells.

As we defined CD8 T-cell responses by the ability to respond to stimulation by HLA class I-presented, autologous HIV epitopes, we did not analyze terminally exhausted T cells that completely lost their capability of reacting with production of any analyzed functional marker to antigen exposure. This complicates comparison to studies using tetramer binding when defining HIV-specific immune responses and leads to potential underestimation of the magnitude of CD8 T-cell responses. However, peptide stimulation has for a long time been the method of choice in the field, and the use of tetramers limits the study of HLA-restricted responses to those available.

The median frequencies of IL-2-producing HIV-specific CD8 T cells were higher in the HLA-B*57-positive group in both early and late chronic infection, indicating improved proliferative capacity. Consistent with previous studies, we found no difference in the ability of HIV-specific cells to degranulate (20). Nevertheless, a longitudinal trend toward a higher frequency of CD107a-expressing cells in the HLA-B*57-negative patient group confirms the importance of vesicle content over the ability to degranulate upon target cell recognition (20). In agreement with this, the HLA-B*57-positive group displayed significantly more cells with multiple functional ability, including perforin, TNF, granzyme A, and granzyme B, in early chronic infection. Furthermore, the HLA-B*57-negative group progressively further lost the ability to produce GrzA as well as cells with the ability to produce GrzA, perforin, and TNF.

We found pronounced discrepancies in inhibitory receptor expression when comparing HLA-B*57:01-restricted HIV epitope-specific CD8 T cells to responses restricted by other HLA class I alleles. During the early stages of chronic infection, HLA-B*57:01-positive patients showed lower frequencies of CD8 T cells expressing the inhibitory receptors TIGIT and PD-1. The frequency of inhibitory receptor-expressing cells increased over time for all patients and reached similar levels in the late stage of infection, when all patients show signs of disease progression. Analysis of the simultaneous expression patterns of single cells revealed that the differences are strongly driven by TIGIT and PD-1 double-positive cells among the HIV epitope-specific CD8 T-cell responses of HLA-B*57-negative patients. Conversely, the number of TIGIT and PD-1 double-negative cells is significantly higher among HLA-B*57:01-restricted CD8 T-cell responses, suggesting reduced exhaustion in this cell population. When linking the frequency of TIGIT and PD-1 double-positive cells to clinical measures of disease progression, we confirmed a strong negative correlation with CD4% and CD4/CD8 ratio as well as a weaker positive correlation with viral load. This discrepancy is partially caused by a lower viral load but also suggests that the frequency of TIGIT and PD-1 double-positive CD8 T cells might be even more closely related to the deterioration of the immune system, in particular the reduced quality and quantity of CD4 T-cell help (36, 37).

The association of combined expression of multiple inhibitory receptors with diminished function of CD8 T cells (38) and the protective effect of the HLA-B*57:01 allele (39, 40) are well established, and our study links those phenomena. While numerous mechanisms for delayed disease progression in HLA-B*5701-positive patients have been suggested (9, 18, 23, 41), a complex network of several contributing factors is likely to confer the protective effect. Our findings suggest a detrimental influence of early TIGIT and PD-1 coexpression in HIV infection. Earlier emergence of this cell population is associated with loss of lytic granule loading at later stages, disadvantageous changes to the polyfunctionality pattern of CD8 T cells, and a potentially inadequate pool of progenitor cells among HIV-specific cells. Cross-sectional studies showed that expression levels of TIGIT on CD8 T cells increase with HIV disease progression (26). However, elite controllers maintain lower TIGIT (26) and PD-1 (42) expression levels on CD8 T cells. These findings are in line with the exacerbation of T-cell exhaustion with increased antigenic load in infection models (43), and our data confirm increased frequencies of TIGIT and PD-1 double-positive cells among HIV epitope-specific CD8 T-cell responses. Similar to our findings, an association between PD-1 and CD38 double-positive CD8 T cells and clinical measures of disease progression has been reported, strengthening the evidence linking T-cell activation/exhaustion in early HIV infection to the rate of disease progression (37). Most inhibitory receptors, especially PD-1 and KLRG-1, are individually also used as markers for T-cell activation in acute infection, while their combined expression during chronic disease is linked to exhaustion and loss of functionality. Studies of checkpoint blockade in cancer therapy show synergy between the inhibitory receptors TIGIT and PD-1 (44, 45), further supporting the role of this cell population.

The longitudinal results indicated a delay in progressive immunological changes in HLA-B*57-positive compared to those in HLA-B*57-negative patients. Given recent findings of a pool of progenitor T cells maintaining the exhausted T-cell population, analysis of the responsible transcription factors, proliferation, and turnover of exhausted HIV-specific T-cells is of interest. In our study, HLA-B*57-resticted T-cell pathogenesis was linked to a transition in the expression of T-bet and Eomes, from T-bet^hi^ Eomes^dim^ to T-bet^dim^ Eomes^hi^, which we and others have previously linked to a CD8 T-cell exhaustion profile (25). Future studies will be needed to clarify whether the longitudinal differences might also be explained by a different quality of the progenitor pool and thereby homeostasis, leading to an earlier host-pathogen “stalemate” in HLA-B*57-negative patients (reviewed in references 24 and 46). This could also explain the maintained capacity of HLA-B*57-positive patients to generate new responses to mutant epitopes (19, 23).

Studies of the immunopathological events that occur during HIV infection remain important to increase the quality of life of HIV-infected individuals. Since T-cell exhaustion strikes the entirety of a patient’s T-cell population and the inhibitory networks probably include all hematopoietic cell types, targeted interference with these networks might improve not only the direct HIV burden but also non-AIDS complications and other immune-related treatments like vaccination. In addition, the higher risk of cancer development due to HIV infection with the rise of immunotherapies as cancer treatments increases the likelihood of HIV patients facing those therapies, and knowledge about how HIV influences T-cell exhaustion might aid in the choice of regimen. Details of T-cell exhaustion are likely to influence aspects such as side effects and efficacy of checkpoint blockade or chimeric antigen receptor (CAR) T cells generated from autologous T cells. By combining multiparametric immunology and advanced statistical bioinformatics, we conclude that the combined expression of the inhibitory receptors TIGIT and PD-1 early in chronic HIV infection is likely to diminish the efficacy of the HIV-specific CD8 T cells. This multiplex approach is of particular interest for future clinical, therapeutic vaccine, or cure studies in which multiple markers are combined to understand pathological mechanisms of the T-cell repertoire in HIV-infected subjects.

## MATERIALS AND METHODS

### Study subjects.

Twelve HIV-infected subjects (P1 to P12) were selected from the San Francisco-based cohort OPTIONS at the University of California (47) (Tables 1 and 2). Patients were monitored for up to 7 years from early infection, where the time of infection was estimated as the midpoint between the last reported negative test and the first positive test (9). Six subjects (P1 to P6) carried the HLA B*57:01 allele (9). The other six subjects (P7 to P12), not carrying the HLA allele B*57, have been described in an article linking early HIV-specific responses against HIV Gag to slower disease progression (12). Healthy control samples (*n* = 9) were selected based on the presence of CMV- or Epstein-Barr virus (EBV)-specific responses, as well as age (median, 33 years; interquartile range [IQR], 26 to 33 years) and gender (89% males). The University of California, San Francisco (UCSF), Committee on Human Research (IRB no. 10-00301) and the Regional Ethical Council in Stockholm, Sweden (2008/1099-31, 2016/1314-32), approved this study, and all patients provided written informed consent.

### RNA extraction, cDNA synthesis, PCR amplification, and sequencing.

Longitudinal plasma samples were stored at –80°C and subsequently processed. HIV *gag* p24 single genome sequences for the HLA-B*5701 subjects (P1 to P6) were obtained as previously described (9). For the control subjects (P7 to P12), viral RNA was extracted from 1 ml plasma with a QIAamp viral RNA minikit, and the entire viral RNA extraction was used for cDNA synthesis. cDNA synthesis was performed using a Superscript III system (Invitrogen) with gene-specific primers using a limiting-dilution digital nested-PCR, single-genome sequencing (9). Sequencing of the PCR products was performed at the sequencing core facility of the University of Florida on an ABI3700 system, under IRB approval no. 258-2012. Sequences were assembled with Geneious 5.6 software created by Biomatters (Auckland, New Zealand). Chromatograms were manually examined for the presence of double peaks indicative of at least two templates per sequencing reaction, and these genomes were discarded.

### Sequence analysis.

Subject-specific alignments were manually obtained with the freely available BioEdit software developed by Tom Hall. For each data set, mean nucleotide divergence and diversity were estimated at each time point, and standard errors were calculated by bootstrapping (500 replicates), with standard errors inferred from 500 bootstrap replicates. Calculations were carried out with MEGA5 software (48). The phylogenetic signal in each data set was investigated by likelihood mapping (49). Likelihood mapping analyses were performed with the program TREE-PUZZLE (50) for each data set by analyzing 10,000 randomly chosen quartets. The presence of potential intrahost recombinant sequences was investigated with a previously published PHI test-based algorithm (51). Calculations were performed with the SplitsTree package version 4.8.

### Flow cytometry.

Flow cytometry was performed as previously described (26). Briefly, peripheral blood mononuclear cells (PBMCs) were isolated using Ficoll-Paque density gradient separation and stored in liquid nitrogen. The PBMCs were thawed, washed, and left to rest for 6 h. The cells were stimulated with autologous HLA-restricted HIV Gag peptides or optimal CMV or EBV peptides (JPT Innovative Peptide Solutions) in the presence of monensin (BD Bioscience), brefeldin A (BD Bioscience), and CD107a antibody for 10 h. Thereafter, the cells were washed, extracellularly stained (exhaustion panel), permeabilized (human FoxP3 buffer set; eBioscience), and intracellularly stained before analysis on the flow cytometer (LSR Fortessa; BD). For lineage identification and determination of differentiation phenotypes, CD14/CD19 V500 (M5E2/HIB19; BD Bioscience), CD27 BV785 (O323; Biolegend), CD3 APC-H7 (SK7; BD Bioscience), CD45RO BV605 (UCHL1; BD Bioscience), CD4 BV711 (OKT4; Biolegend), or PE-Cy5.5 (S3.5; Invitrogen) and CD8 Qd565 (3B5; Life Technologies) were used in both panels. We stained for T-bet phycoerythrin (PE) (4B10; Biolegend) and eomesodermin (Eomes) PE-eF610 (WD1928, eBioscience) in both panels due to their central role in effector function and survival of memory T cells, as well as CD107a PE-Cy5 or PE-Cy7 (H4A3; BD Bioscience) and IFN-γ AF700 (B27; BD Bioscience) for identification of CD8 T-cell responses after peptide stimulation. A panel focused on inhibitory receptors (exhaustion panel) included staining of KLRG-1 AF647, CD160 AF488 (BY55; BD Bioscience), PD-1 BV421 (EH12.2H7; Biolegend), 2B4 PE-Cy5 (C1.7; Beckman Coulter) and TIGIT PE-eF710 (MBSA43; eBioscience). The second panel (function panel) included GrzB PE-Cy5.5 (GB11; BD Bioscience), IL-2 APC (MQ1-17H12; BD Bioscience), GrzA AF488 (CB9; Biolegend), perforin BV421 (B-D48; Biolegend), and TNF PE-Cy7 (MAb11; BD Bioscience) for a detailed analysis of effector functions, including degranulation capacity, loading of cytotoxic granules, and cytokine production.

### Data analysis and statistics.

Data were analyzed with FlowJo LLC software (10.1r1), SPICE (52), GraphPad PRISM (version 6.0d), and R scripts (custom tailored in collaboration with the research group of Ole Lund, DTU, Copenhagen). To compare the combined expression of inhibitory receptors on epitope-specific CD8 T cells between the patient groups, we used permutation tests and Student´s *t* test provided in SPICE. Cross-sectional comparisons in GraphPad PRISM were made with Mann-Whitney tests; for correlations of total (memory) CD8 T cells with duration of infection and clinical parameters, Spearman´s rank correlations were determined. To avoid bias toward patients with multiple available responses, mixed-effects models were calculated (Table 5) with Stata v16.1 software (StataCorp LLC, College Station, TX) when comparing HIV epitope-specific responses. If not indicated otherwise, linear mixed-effects models were used. Due to the scarcity of the cell population depicted, a zero-inflated negative binomial (ZINB) regression was used for the statistical analysis shown in Fig. 6F. All results of mixed-effects models are shown in Table 5.

### Data availability.

GenBank accession numbers for the sequences for subjects P1 to P6 are JX234575 to JX235332. GenBank accession numbers for the sequences for subjects P7 to P12 are MT274756 to MT275450.
